# YAP/TAZ dull the STING of aging

**DOI:** 10.20517/jca.2022.33

**Published:** 2022-08-05

**Authors:** Jamie Francisco, Dominic P. Del Re

**Affiliations:** Department of Cell Biology and Molecular Medicine, Cardiovascular Research Institute, Rutgers New Jersey Medical School, Newark, NJ 07103, USA.

Cellular senescence is a concerted process that involves a stable cell cycle arrest despite continued metabolic activity, and the development of a pro-inflammatory response known as the senescence-associated secretory phenotype (SASP)^[1]^. Aging and senescence have long been associated, and studies employing senolytic approaches, i.e., the targeted removal of senescent cells, have demonstrated a causal role for their actions in aging-related phenotypes in various tissues^[2]^. Presumably, due to the nature of SASP, this process is largely non-cell autonomous and involves paracrine effects on neighboring cells to promote organ dysfunction.

Despite our burgeoning knowledge regarding the role of senescence in aging, fundamental questions remain including: which cells first undergo senescence during physiological aging? What is the initial trigger to induce cellular senescence in normal aging? What pathways are amenable to, or suppressive of, physiological aging, and are up- or downregulated during lifespan?

A recent study from Sladitschek-Martens *et al.* published in *Nature*, tested the possibility that altered mechanosensing of the extracellular environment, i.e., the extracellular matrix (ECM), is a signal for physiological aging^[3]^. The transcriptional coactivators and end effectors of the Hippo signaling pathway, YAP and TAZ, are established mediators that link mechanosensation to changes in cell behavior through the regulation of transcriptional programs^[4,5]^. To explore the hypothesis that YAP/TAZ mediate effects of aging, the authors first performed a series of experiments employing single-cell RNA-seq data that indicated the downregulation of a YAP/TAZ activation signature gene set in dermal fibroblasts of old mice. This pattern of depressed YAP/TAZ activity was also observed in other stromal cells (e.g., kidney fibroblasts) as well as contractile cells (cardiomyocytes, vascular smooth muscle cells) but not in epithelial cells, hepatocytes, or lymphocytes, indicating cell-type specificity. To corroborate these findings, YAP/TAZ nuclear (active) staining demonstrated a progressive decline with age in the skin and aorta wall, as were phosphorylated myosin light chain and phosphorylated focal adhesion kinase, indicating cytoskeletal tension and integrin engagement were also depressed with age.

To test a causative role for YAP/TAZ in physiological aging, Sladitschek-Martens *et al.* conditionally deleted YAP/TAZ in fibroblasts *in vivo*^[3]^. Depletion of YAP/TAZ in young mice reduced dermal fibroblast number and phenocopied aged skin in control mice. Targeted deletion of YAP/TAZ in vascular smooth muscle cells elicited aortic dissection, rupture, and death several weeks after Cre induction, thereby accelerating aging-associated pathology. Importantly, the add back of constitutively active YAP^S127A^ using a fibroblast selective inducible transgenic model was sufficient to rescue normal aging of the skin. The authors also leveraged a fibrillin mutant mouse strain (Fbn1^C1039G/WT^) to directly modulate the ECM, resulting in decreased nuclear YAP/TAZ and an early onset aging phenotype of the vascular wall. Similar to the genetic studies in dermal fibroblasts, FBN1 mutant-induced aging-associated tissue breakdown was rescued by inducible smooth muscle cell expression of YAP^S127A^. These elegant studies provide strong evidence that dysregulation of YAP/TAZ mechanotransduction contributes to murine physiological aging.

To further substantiate YAP/TAZ regulation of cellular senescence, transcriptome profiling in freshly isolated dermal fibroblasts from young mice demonstrated increased SASP genes as well as increased β-gal expression, both hallmarks of senescence, in YAP/TAZ deficient cells. On the other hand, supplementing YAP to fibroblasts cultured from old mice led to suppression of SASP and β-gal positivity. Similarly, inhibition of integrins or RhoA, which are established YAP/TAZ activators, triggered a senescent phenotype, further supporting the hypothesis that mechanotransduction is a critical input to regulate these cellular responses.

cGAS-STING signaling modulates innate immune responses and has been previously implicated in the regulation of senescence^[6]^. Through multiple complementary approaches, Sladitschek-Martens *et al.* showed that YAP/TAZ suppressed cGAS activation in several cell and tissue types, and involved the inappropriate release of genomic DNA into the cytosol^[3]^. Because the induction of SASP genes in YAP/TAZ deficient cells was sensitive to cGAS and STING inhibition, the authors utilized triple mutant mice in which YAP/TAZ and STING were inactivated to rescue the senescent aging phenotype in skin, aortic wall, and kidney, thereby directly linking the YAP/TAZ and cGAS-STING pathways *in vivo*.

How does YAP/TAZ restrain cGAS-STING? The authors astutely recognized a relationship between decreased YAP/TAZ activity and distorted nuclear architecture in old cells. Moreover, the addition of active YAP rescued abnormal nuclear structure caused by aging. Additional screens revealed that YAP/TAZ directly promote the expression of two key factors that maintain proper nuclear envelope integrity, lamin B1 and ACTR2, the latter a component of the ARP2/3 complex required for actin filament nucleation and maintenance of the peri-nuclear actin cap^[7]^. Consistent with this hypothesis, depletion of ACTR2 elicited nuclear deformation, cGAS activation, and SASP gene expression.

Senescent cell accumulation is linked to aging, and senolytic approaches ameliorate aging-related tissue degeneration^[1,2]^. The study from Sladitschek-Martens *et al.* elegantly linked physiological aging and cellular senescence to a decline in mechanotransduction mediated by decreased YAP/TAZ activity^[3]^. These findings demonstrate that YAP/TAZ is a critical upstream modulator of nuclear integrity and functions in young cells to keep cGAS-STING in check to prevent the aging phenotype [Figure 1].

Of note, this newly identified YAP/TAZ mechanism was not generalizable to all cell types examined, and appears to manifest primarily in stromal and contractile cells. For example, decreased YAP/TAZ activity was observed in cardiomyocytes, however, the potential implications of this response in the context of physiological aging remain to be fully explored. Cardiomyocytes are thought to undergo senescence^[8]^, yet the molecular underpinnings that regulate this process during aging remain unclear, and it will be of interest to determine whether the mechanism proposed by Sladitschek-Martens *et al.* holds true in this multinucleated and terminally differentiated cell type^[3]^. Another avenue for pursuit in future studies will be deciphering the upstream signal(s) that precipitates decreased YAP/TAZ activity during physiological aging. This study raises the intriguing possibility that altered ECM properties and/or maladaptive ECM-cell interactions are responsible for aberrant mechanotransduction thus initiating this cascade under physiological conditions. Interestingly, decreased mechanical force was shown to antagonize YAP nuclear translocation through altered nuclear pore accessibility^[9]^, and may represent an additional layer of regulation that serves to inhibit the nuclear abundance of YAP/TAZ in aging. Whether canonical Hippo signaling is modulated and contributes to the suppression of YAP/TAZ in aged cells also remains to be clarified. Finally, addressing how this mechanism might integrate with other proposed triggers of cellular senescence, such as telomere shortening, mitochondrial dysfunction, or impaired autophagy, should provide valuable insight. Nevertheless, the study by Sladitschek-Martens *et al.* has revealed a novel and important mechanism linking YAP/TAZ and cGAS-STING to the induction of cellular senescence and physiological aging, which may represent new therapeutic targets to improve life and healthspan^[3]^.

## Figures and Tables

**Figure 1.**
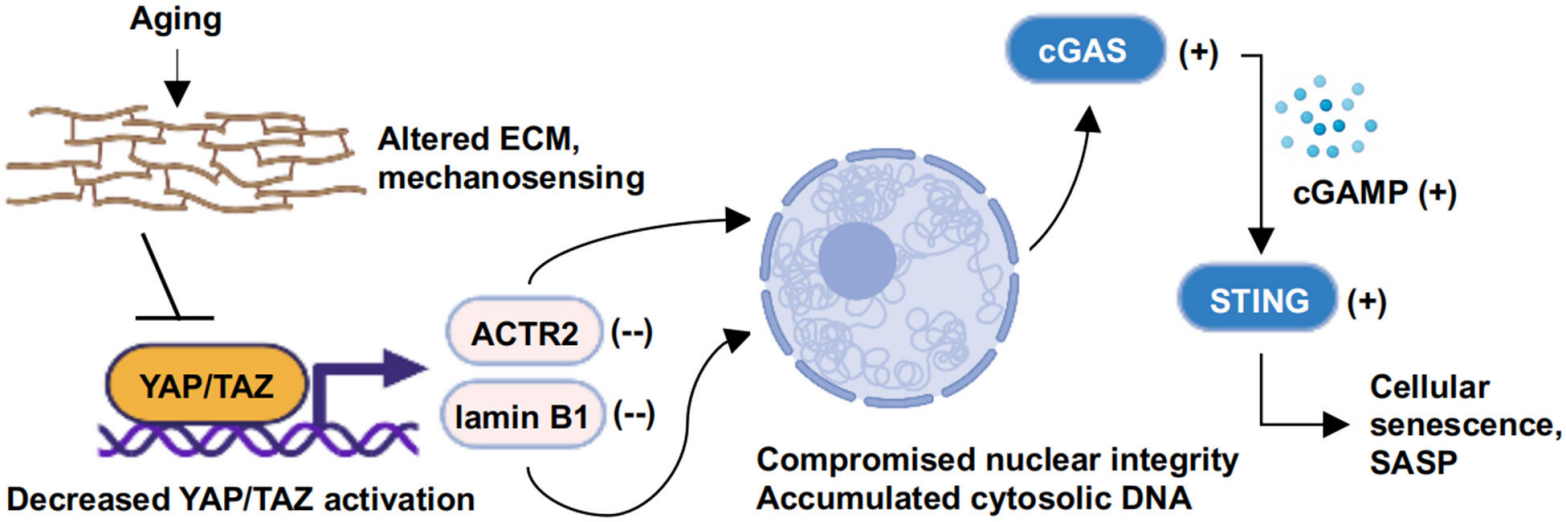
A scheme representing YAP/TAZ mediated repression of cellular senescence. Under physiological aging conditions, mechanotransduction and subsequent YAP/TAZ activity are decreased, leading to the decreased expression of ACTR2 and lamin B1, two direct transcriptional targets of YAP/TAZ. The downregulation of ACTR2 and lamin B1 elicits a decline in the structural integrity of the nuclear envelope and the release of genomic DNA into the cytosol, which triggers the activation of cGAS-cGAMP-STING signaling. cGAS-STING was shown to mediate hallmarks of cellular senescence, including SASP, during aging. Created using BioRender.com.
